# Assessment of dynamic stability and identification of key tasks, inertial sensors, and parameters in patients with bilateral and unilateral vestibulopathy: investigation in a semi-standardized environment

**DOI:** 10.1186/s12984-026-01933-8

**Published:** 2026-03-12

**Authors:** Gautier Grouvel, Thomas Zimmermann, Samuel Cavuscens, Anissa Boutabla, Jean-François Cugnot, Raymond van de Berg, Nils Guinand, Stéphane Armand, Angélica Pérez Fornos, Julie Corre

**Affiliations:** 1 Division of Otorhinolaryngology Head and Neck Surgery, Geneva University Hospitals and University of Geneva, Geneva, Switzerland; 2 Kinesiology Laboratory, Geneva University Hospitals and University of Geneva, Geneva, Switzerland; 3 Centre of Research on Skeletal Muscle and Movement, Geneva University and Geneva University Hospitals, Geneva, Switzerland; 4 University of Applied Sciences and Arts Western Switzerland, HES-SO, Lausanne, Switzerland; 5 Clinical Neurosciences Department, Neurorehabilitation Department, Geneva University Hospitals, Geneva, Switzerland; 6 Division of Balance Disorders, Department of Otorhinolaryngology and Head and Neck Surgery, Maastricht University Medical Center+, Maastricht, The Netherlands

**Keywords:** Vestibulopathy, Inertial sensors, Daily living tasks, Stability, Movement, Assessment

## Abstract

Monitoring the functional status of patients with bilateral vestibulopathy (BV) or unilateral vestibulopathy (UV) in an ecological environment remains a challenge, as current tests are performed in specialized laboratories and do not reflect patients’ limitations and symptoms. This study aimed to evaluate the overall movement patterns of patients using inertial measurement units (IMU) in a semi-standardized environment that replicated daily life situations. Nineteen BV patients, 20 UV patients, and 20 healthy subjects were recruited to perform 15 daily-living tasks. The intensity, smoothness, and stabilization parameters were calculated from IMU data. The relevant tasks were selected on the basis of their duration and perceived difficulty. Principal component analysis (PCA) was used to identify the variables that contributed most to the variance in the dataset, and their discriminating ability between groups was assessed using Kruskal-Wallis tests. Tasks that minimize or deprive compensatory sensory inputs (e.g., proprioceptive or visual cues), thereby increasing reliance on vestibular information, proved to be the most discriminative between patients with bilateral vestibulopathy and healthy subjects with IMUs placed on the feet (e.g., putting on pants, walking on uneven ground, on inclined plane, in the dark, on a wood beam, or with head turns). BV and UV patients exhibited reduced movement intensity compared to healthy subjects, which can reflect different compensatory stabilisation strategies. This study demonstrated the feasibility of objective functional status assessment using IMUs and provides a basis for the development of rapid clinical protocols, to assess outcomes for rehabilitation therapies.

**Supplementary Information:**

The online version contains supplementary material available at 10.1186/s12984-026-01933-8.

## Introduction

Bilateral and unilateral vestibulopathy (BV and UV, respectively) can lead to drastic functional impairments [1] and reduced quality of life [2, 3]. Chronic imbalance/unsteadiness is one of the main symptoms [1], particularly during tasks where compensatory sensory inputs (vision, proprioception) are limited, such as walking in the dark or on uneven ground. Although, current clinical diagnostic tests such as the video Head-Impulse test (vHIT) [4], caloric test, rotatory chair testing or cervical Vestibular Evoked Myogenic Potentials (cVEMPS) [5] are used to assess vestibular reflexes, they do not adequately reflect the severity of patients’ symptoms [1] and their limitations in daily life [2, 3]. Moreover, objective monitoring of their functional status has not yet been implemented in clinical practice.

To our knowledge, only a limited number of studies have investigated movements in patients with vestibulopathy during various ecological, daily life tasks (such as putting on trousers, putting on shoes, walking, or walking on an inclined plane). One study [6] assessed head movements in UV patients during daily life tasks. However, this study was limited to analysing head movements, without studying overall body movement. Moreover, there is an evident lack of data on this topic for BV patients.

Studies analysing gait and balance in BV and/or UV patients highlighted altered spatio-temporal parameters compared with healthy subjects [7–10]. They also reported increased instability and variability during complex tasks [9, 11] such as walking with eyes closed or walking with a narrow base of support. BV patients also exhibited different head stabilisation strategies, characterized mainly by increased rigidity of the head and trunk compared with healthy subjects [12, 13]. However, these findings were obtained in standardized and supervised laboratory environments, which may not reflect patients’ movement behaviour in daily life conditions.

More recently, wearable sensors combined with machine-learning approaches have been used to discriminate gait patterns between patients with various peripheral vestibular disorders. For instance, Jabri et al., [14] showed that a single inertial measurement unit (IMU) placed on the left arm could reliably identified affected participants while walking with eyes closed. This particularly intriguing approach nevertheless raises methodological questions, particularly with regard to the location of the best sensor, and the patient inclusion criteria for population comparison which may limit the generalisability of the results.

All the above highlights the need to develop accurate, rapid, and effective tests to monitor the evolution of patients’ functional status over time, but also to evaluate current and future rehabilitation therapies (such as vestibular implants), which remains a major challenge [15–17]. It is also necessary to assess patients’ movement when they perform tasks close to daily life in order to identify those that are the most relevant for monitoring their functional status, ideally without the need of specialised or complex laboratory equipment.

Therefore, this study aimed to evaluate the overall movement of patients with chronic BV and UV and compare these movements to those of healthy subjects in a semi-standardized environment. Such an environment replicates daily life situations, while being supervised by two operators.

Based on the results of previous studies [7–10, 12, 13] and complaints expressed by patients, we hypothesize that (1) they will experience greater difficulties in performing tasks that minimize or deprive compensatory sensory inputs (e.g. walking on uneven ground or walking in the dark), as these conditions reduce the usefulness of alternative sensory modalities and thereby increase reliance on vestibular function. (2) They will also need more time to perform tasks. (3) They will have limited movement compared to healthy subjects, and (4) The most relevant sensor positions will be the trunk and the head.

## Methods

### Study design

Wearable IMUs were placed on different anatomical segments of the participants’ bodies to measure the intensity, stability, and regularity of their movements. Principal component analysis (PCA) and discriminant analysis were then performed. These analyses identified the most relevant tasks and sensor positions for a rapid and effective functional assessment of patients’ movements in an environment that can be transposed to rehabilitation facilities or home-based patient monitoring.

## Population

The participants of this study were the same as those previously described [18]. It included a group of 19 patients with bilateral vestibulopathy (BV) (11 females, mean (sd), age: 60.2 (11.6) years, height: 169.1 (8.6) cm, weight: 71.5 (13.7) kg, BMI: 24.9 (3.5) kg/m^2^), a group of 20 patients with unilateral vestibulopathy (UV) (10 females, 9 left affected, mean (sd), age: 59.5 (5.5) years, height: 173.4 (10.0) cm, weight: 74.5 (13.5) kg, BMI: 24.7 (3.4) kg/m^2^), and a group of 20 healthy subjects (HS) (10 females, mean (sd), age: 57.9 (5.3) years, height: 172.1 (8.4) cm, weight: 72.1 (13.5) kg, BMI: 24.3 (4.1) kg/m^2^).

BV patients were previously diagnosed according to the guidelines of the Classification Committee of the Bárány Society [19]. These criteria include the presence of unsteadiness and/or oscillopsia when walking or standing, no symptoms while sitting down or lying down, bilaterally reduced or absent vestibulo-ocular reflex (reduced horizontal VOR gain (< 0.6) as measured by the vHIT; and/or reduced caloric response (< 6 °/s); and/or reduced horizontal VOR gain (< 0.1) during sinusoidal stimulation on a rotatory chair) – raw data of the clinical vestibular tests are present in Supplementary Materials S1.

Because no consensus diagnostic criteria currently exist for chronic UV, patients in the UV group were included based on lateral-canal vHIT findings, consistent with unilateral VOR impairment. Inclusion required: a lateral semicircular canal gain < 0.6 on the affected side, a gain > 0.8 on the contralateral side, symptom duration ≥ 3 months, and no evidence of other otologic or neurologic disease – raw data of the clinical vestibular tests are present in Supplementary Materials S1.

Finally, all HS were screened through a comprehensive structured interview, which confirmed no history of dizziness, vertigo, or imbalance, no hearing loss or otologic disease, no neurologic conditions, no prior exposure to ototoxic medication, no other medical conditions affecting vestibular or balance function. vHIT was not performed in HS, as their asymptomatic status and absence of risk factors were confirmed through detailed history. This approach is consistent with common practice in vestibular research when laboratory testing is not required for control group classification.

All study participants were over 18 years of age and provided their written informed consent.

## Measurement protocol

During their visit, participants performed a series of 15 tasks representative of daily living activities and difficulties typically encountered by patients [6, 20, 21], in a semi-standardized environment simulating ecological conditions but under supervision. This study was part of a larger protocol, and participants were equipped with a set of wearable sensors (IMUs, plantar pressure insoles, and eye trackers). After completing each task, the participants were asked to rate the difficulty of the task as easy, medium, difficult, or impossible. Each session was video recorded by an operator, with the patient facing away from the camera, to enable visual analysis of the movements.

The IMUs were secured with elastic bands so that they remained fixed to the body segments. Care was taken to position the sensors consistently across participants to ensure reproducible data. The complete protocol is described in detail in a separate article [22].

The tasks performed by the participants are presented in Table 1.

**Table 1:** Description of tasks

Tasks	Description
Bed	The subject lies on his/her back and flat on the bed, remains still for 3 s, and then gets up from the bed.
Pants	Standing up, the subject puts a pair of loose pants on, one leg at a time, and then takes them off.
Shoes	Without sitting or kneeling, the subject removes his/her shoes one after the other and then puts them back on.
Sorting	The subject sorts plastic crockery from a storage box onto a shelf as quickly as possible according to colour and size.
Heavy load	The subject must carry a heavy load (5 kg) for 10 m, turn around, change hands and return to the starting point (total: 20 m).
Bus	The subject must press the button to open the door, get on the bus, sit down for a few moments, press the stop button, get up, and get off the bus.
Stairs	The subject must climb 8 straight steps (28 cm width steps, 17 cm spaced from each other) and descend 6 spiral steps (25 cm width steps, 18 cm spaced from each other). If possible, without holding the ramp.
Uneven ground	The subject walks on an unstable cobbled path (15 m).
Tray	The subject must carry two glasses of water on a tray over 12 m without dropping them. The glasses were filled to around 90% of their capacity, with a total weight of approximately 350 g.
Walk	The subject walks in a straight line (12 m) at her/his most comfortable pace.
Stepladder	Subject climbs 5 steps-ladder then comes off (8 cm width steps, 21.3 cm spaced from each other).
Wood beam	The subject walks on a 4.5-m (15 cm width) wooden beam, turns around, and walks back.
Inclined plane	The subject climbs a ramp (25 m – 15°), turns around, and descends it again, the first half of the descent being made with eyes closed.
Picture recognition	Pictures of scenes (x 3) from daily life are hung on the windows of the room (distance: ≈2 m, height: eye level). The subject looks at the pictures (29.7 × 21 cm) while walking (1 round trip: 20 m) and tells the examiner what he/she has seen, giving as much detail as possible. The subject must not stop while walking.
Walk in the dark	Subject walks with custom-made darkening glasses (welding glasses) on level ground for 12 m.

**Fig. 1:**
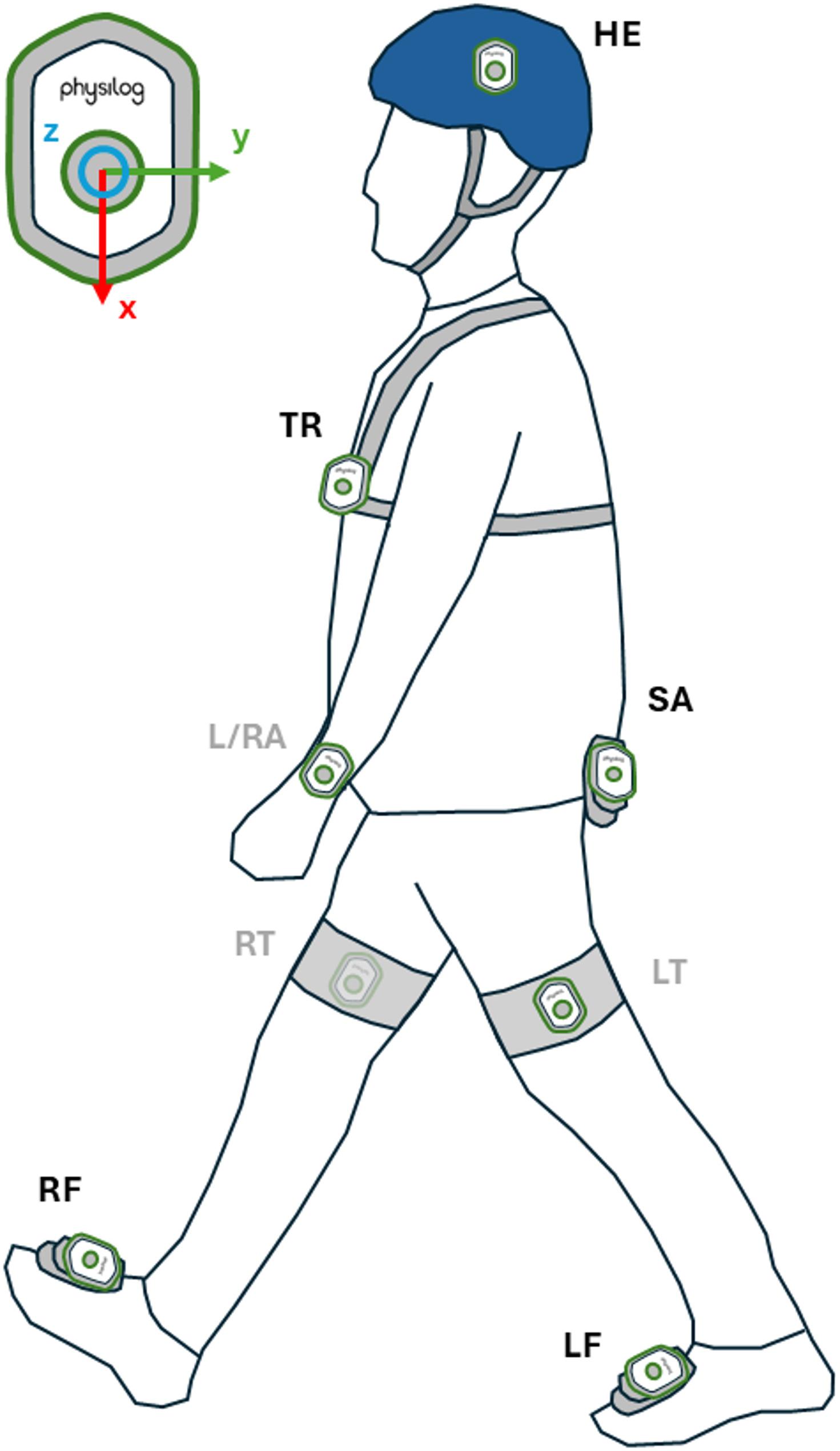
Schematic illustration of the position of the IMUs on the body, as well as their coordinate system. [*HE* Head, *TR* Trunk, *SA* Sacrum, *L/RA* Left/Right arm, *LT* Left Thigh, *RT* Right Thigh, *LF* Right Foot, *RF* Right Foot. Gray names: IMUs not used in the analysis]

In this study, we focused solely on the analysis of IMU data. The participants were equipped with 9 IMUs (Physilog6S, MindMaze, Lausanne, Switzerland) placed on the head, trunk, pelvis (sacrum), left and right wrists, left and right thighs, and left and right feet (Fig. 1). They recorded 3-Dimensional (3D) linear accelerations (128 Hz, ± 16 g) and 3D angular velocities (128 Hz, ± 2000 °/s). Throughout the session, IMUs were controlled by a mobile app to start and stop data recording for the corresponding task. No calibration task was recommended by the manufacturer’s documentation, as the IMUs have already been calibrated in the factory.

## Data processing

**Fig. 2:**
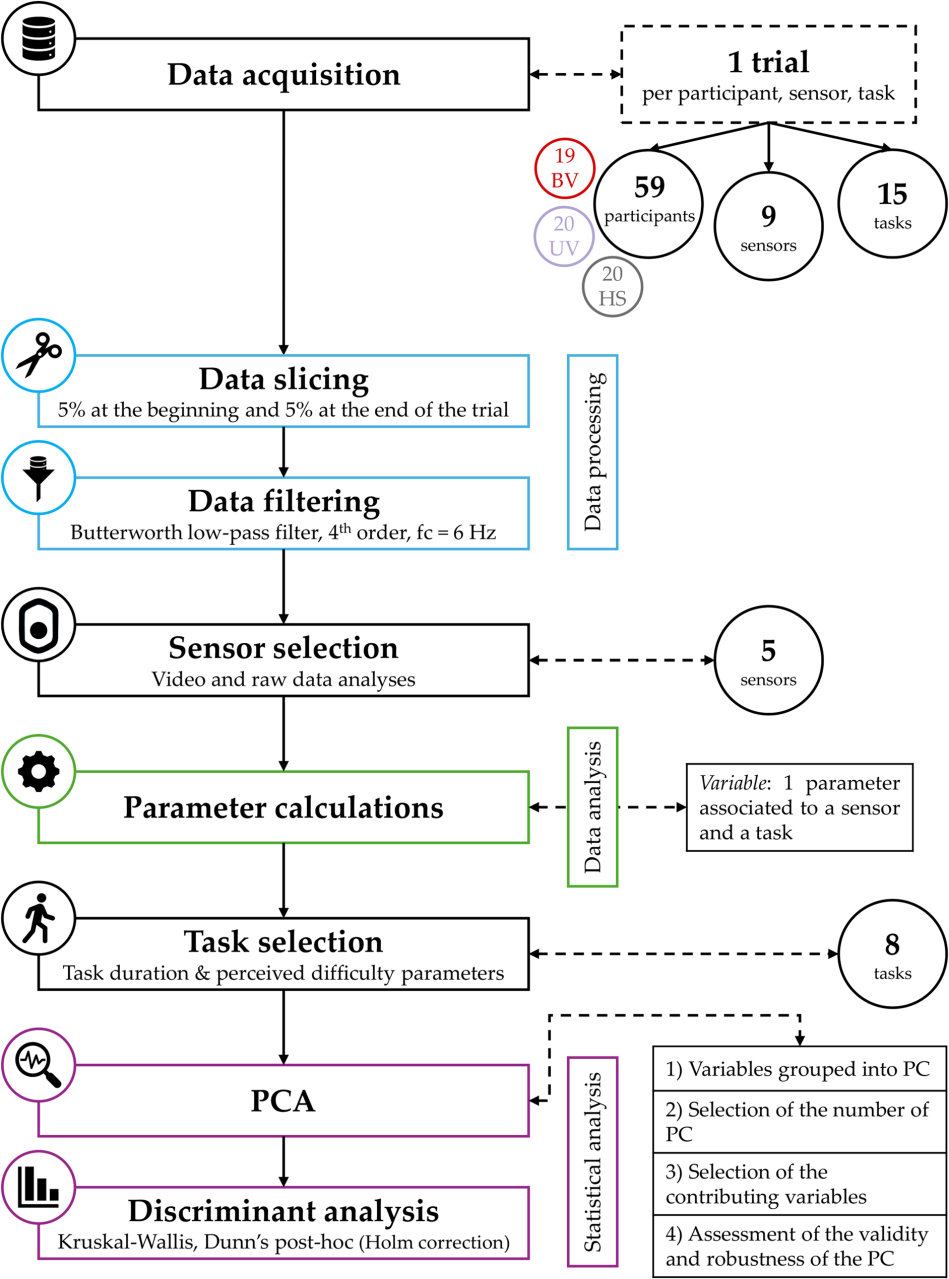
Flowchart for the data processes and analyses. [*BV* Bilateral vestibulopathy *patients*, *UV* Unilateral vestibulopathy *patients*, *HS* Healthy subjects, *fc* Cut-off frequency, *PCA* Principal component analysis, *PC* Principal component]

The raw data, i.e., angular velocities and linear accelerations, were processed separately by participant, by task, and by IMU: referred to as a *trial*. The data were extracted and analysed using a custom-made MATLAB code (MATLAB R2022b, The MathWorks Inc., MA, US). For each trial, the initial 5% and final 5% of the total duration were removed to eliminate the initiation and termination phases, in order to minimize any measurement artefacts. The raw data were then filtered using a 4th order Butterworth low-pass filter, with a cut-off frequency of 6 Hz [23]. The flow chart of data processing is presented in Fig. 2.

### Data analysis

After initial video analysis and first exploratory analysis of raw signals, we decided to exclude the four IMUs positioned on the wrists and thighs from our analysis. This exclusion was based on two main criteria: for wrist IMUs, video analysis showed that these sensors captured unwanted arm movements unrelated to the tasks or instructions given to participants (e.g., spontaneous movements, variations in arm swing, contact with objects); for thigh IMUs, preliminary visual analysis of the raw data showed that foot IMUs provided more discriminating information for locomotor tasks. Therefore, only 5 IMUs (head, torso, sacrum, left and right feet) were kept for the analysis. Similar configurations of IMUs were used in prior studies, particularly to study multilevel control of upper body movements in children with cerebral palsy [24] or in patients with subacute stroke [25]. This choice also enabled to a significant reduction in the number of parameters calculated, thereby simplifying the analysis. The following parameters were calculated from these IMUs in order to best characterize participants’ movements in terms of intensity, smoothness, and stabilization strategies adopted. Each parameter was calculated from processed data for each participant, each task, and each sensor: referred to as a *variable*.

### Norm of 3D linear acceleration and angular velocity

The norm of the linear acceleration [23] allows for estimation of the intensity of movements of different body segments. This parameter is particularly used in actimetry systems to quantify the intensity level of motor activity [26].

The norm of the angular velocity [23], which is sensitive to the rotational movements of the segments, was also integrated into the analysis.

These two parameters were calculated as follows:

1 $${s}^{3d}=\sqrt{{s}_{x}^{2}+{s}_{y}^{2}+{s}_{z}^{2}}$$

where s^3d^ stands for 3D linear acceleration or 3D angular velocity, s_x, y, z_ stands for the x, y, or z linear acceleration or angular velocity. For these two parameters (Eq. (1)), the root mean square (RMS) value was calculated.

## Sway jerkiness

Sway jerkiness, corresponding to the time derivative of acceleration [27], was calculated as an indicator of the smoothness of postural oscillations [28, 29]. The function associated with 3D jerk is expressed as follows [30]:

2 $$JERK{\text{ }} = \,\frac{1}{2}\,\mathop \smallint \limits_{0}^{t} \left( {\left( {\frac{{dAcc_{x} }}{{dt}}} \right)^{2} + \left( {\frac{{dAcc_{y} }}{{dt}}} \right)^{2} + \left( {\frac{{dAcc_{z} }}{{dt}}} \right)^{2} } \right)dt$$

The RMS value was also calculated for the JERK parameter.

## Attenuation coefficients

Attenuation coefficients, which are calculated between two anatomical segments, enable the assessment of the ability of the patient to attenuate accelerations [31]. They provide information on the strategies used by participants to stabilize their movements [31]. A positive coefficient indicates attenuation of accelerations between the lower and upper segments of the body, whereas a negative coefficient indicates amplification of these accelerations [25, 32]. The attenuation coefficients were calculated between the IMUs of the trunk and head (AC_TH_), sacrum and head (AC_SH_), and sacrum and trunk (AC_ST_), as follows [31]:

3 $$AC_{{ij}} = \,\left( {1 - \frac{{RMS_{j} }}{{RMS_{i} }}} \right) \times 100$$

### Task duration and perception of difficulty

Finally, the duration of the task and the difficulty perceived by the participant were added to the analysis in order to select the most relevant tasks before proceeding with the PCA and statistical test analyses. The duration of the task was also included in the PCA.

### Statistical analysis

Statistical analyses were conducted using R (version 4.5.1) and RStudio (version 2025.05.1 Build 513). The normality of the data was assessed using the Shapiro-Wilk test. Since some variables did not follow a normal distribution, non-parametric tests were used for the entire statistical analysis.

### Task selection

The selection of tasks included in the study was performed using a Kruskal-Wallis test, followed by a Dunn post-hoc test with Holm correction, for both the task duration parameter and the perceived difficulty parameter Table 2. A task was included in the analysis only if the differences between groups were statistically significant for both parameters.

### Principal component analysis

In order to reduce the dimensionality of the dataset and to identify the variables that contribute most to its variance, a PCA was performed [33–35] with all the variables of the three groups for the preselected tasks. This analysis was carried out using the open-source R package *syndRomics* [36]. The methodology followed is based on that described in the study by Torres et al. [36]. PCA allows the initial variables to be grouped into principal components (PC), each representing a portion of the total variance of the dataset. The number of PCs kept was determined by non-parametric permutation tests for the variance accounted for (VAF). For each component, the contributing variables were selected by retaining the 10% highest absolute values of loadings, with a maximum of 10 variables per PC. In order to assess the validity and robustness of the extracted components, a non-parametric bootstrapping procedure was applied over 1000 iterations. A robust PC will have small sensitivity to data variations [37–39].

### Discriminant analysis

The discriminating capacity of the variables identified in the PCs was then evaluated. A comparison between the three groups was performed using Kruskal-Wallis tests, followed, in case of a significant result, by Dunn’s post-hoc analyses with Holm corrections.

## Results

### Task selection

Task duration and perceived difficulty were both statistically significant (Table 2 & Supplementary Materials S2) for 7 tasks: Pants, Heavy load, Uneven ground, Stepladder, Wood beam, Inclined plane, and Picture recognition. Even if only the perceived difficulty parameter was significant between groups, the Walk in the dark task was also added to the analysis as it is a highly relevant task for patients with bilateral vestibulopathy (i.e., it is frequently highlighted by patients and is therefore part of the diagnostic criteria). These 8 tasks were included in the following PCA.

**Table 2:** Results of the Kruskal-Wallis statistical test for task duration and perceived difficulty parameters, followed by the results of Dunn’s post-hoc test with Holm correction, for the tasks selected for subsequent analysis. Results for other tasks are presented in the Supplementary Materials S2. * *p* < 0.05; ** *p* < 0.01; *** *p* < 0.001

Task	Parameter	Kruskal-Wallis (p-value)	Post-Hoc Dunn – Holm correction (p-value)		
			BV - HS	UV - HS	BV - UV
Pants	*Task duration*	0.007**	0.009**	0.039*	0.480
	*Perceived difficulty*	< 0.001***	< 0.001***	< 0.001***	0.179
Heavy load	*Task duration*	0.028*	0.022*	0.188	0.345
	*Perceived difficulty*	0.011*	0.010**	0.358	0.089
Uneven ground	*Task duration*	0.001**	< 0.001***	0.025*	0.264
	*Perceived difficulty*	0.005**	0.004**	0.052	0.328
Stepladder	*Task duration*	0.004**	0.003**	0.301	0.049*
	*Perceived difficulty*	0.022*	0.025*	0.095	0.497
Wood beam	*Task duration*	< 0.001***	< 0.001***	0.014*	0.008**
	*Perceived difficulty*	< 0.001***	< 0.001***	< 0.001***	0.196
Inclined plane	*Task duration*	< 0.001***	< 0.001***	0.001**	0.081
	*Perceived difficulty*	< 0.001***	< 0.001***	0.006**	0.036*
Picture recognition	*Task duration*	0.013*	0.179	0.304	0.019*
	*Perceived difficulty*	0.044*	0.041*	0.232	0.367
Walk in the dark	*Task duration*	0.089	-	-	-
	*Perceived difficulty*	0.006**	0.005**	0.140	0.179

### Principal component analysis

**Fig. 3:**
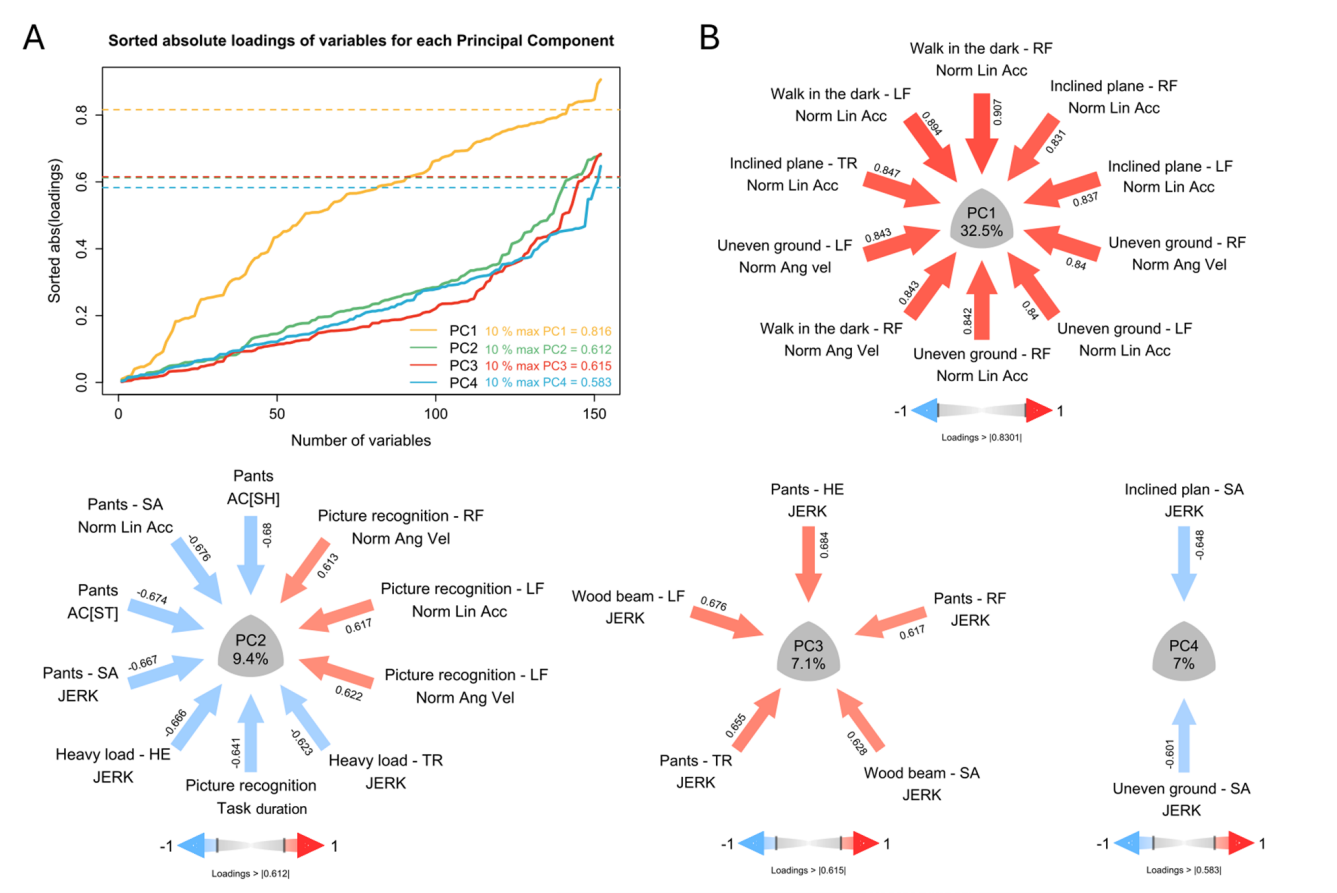
**A**: Sorted absolute loadings of variables for each principal component (PC), to determine the loading threshold for each PC: 10% of the highest absolute values of the loadings, with a maximum of 10 variables per PC. **B**: Principal component analysis (PCA): PC1 to PC4 explain 56% of the total variance. The PCs show the variance of the variables with high loadings on the corresponding PC. The loading values are indicated by numbers placed next to the arrows, whose colour reflects both the magnitude and the sign of the relationship (positive: red, negative: blue). [*PC* Principal component, *RF* Right foot, *LF* Left foot, *TR* Trunk, *SA* Sacrum, *HE* Head, *Norm Lin Acc* Linear acceleration norm, *Norm Ang vel* Angular velocity norm, AC[SH]: Attenuation coefficient of the sacrum and head IMUs, AC[ST]: Attenuation coefficient of the sacrum and trunk IMUs]

Non-parametric permutation tests for the VAF revealed that 7 PCs were relevant for the analysis (Supplementary Materials S3). However, PC6 and PC7 each had a very low explained variance (≤ 4%), and in order to reduce the results, only PCs that together accounted for 50% or more of the variance of the dataset (Fig. 3) were included in the analysis. PCA of 153 variables revealed 4 robustly reproductible PCs (PC1: *r* = 0.968, RMS = 0.096, CC = 0.986, Cattell’s S = 0.952; PC2: *r* = 0.953, RMS = 0.092, CC = 0.953, Cattell’s S = 0.899; PC3: *r* = 0.946, RMS = 0.086, CC = 0.948, Cattell’s S = 0.851; PC4: *r* = 0.957, RMS = 0.078, CC = 0.960, Cattell’s S = 0.892). By retaining only the 10% highest loadings, or a maximum of 10 variables per PC, the selection thresholds for the loadings were as follows: PC1: |loadings| ≥ 0.816, PC2: |loadings| ≥ 0.612, PC3: |loadings| ≥ 0.615, PC4: |loadings| ≥ 0.583 (Fig. 3).

PC1 (32.5% of the variance) was mainly associated with variables of dynamic walking conditions (e.g. Walk in the dark, Inclined plane, and Uneven ground), with a marked contribution from IMUs placed on the left and right feet. One exception was for the variable of the linear acceleration norm parameter for the Inclined plane task, which was associated with the trunk IMU. Overall, the linear acceleration norm had the highest influence on the PC, followed by the angular velocity norm.

PC2 (9.4% of the variance) was characterized mainly by the Pant task, but also by the Picture recognition, and Heavy load tasks. For the Pant and Heavy load tasks, the IMUs located in the upper body (i.e. Head, Trunk, Sacrum), and the acceleration parameters were dominant (i.e. higher loadings). In contrast, for the Picture recognition task, a locomotor task, the duration of the task, and the angular velocity and linear acceleration norm parameters associated with the feet IMUs contributed the most.

PC3 and PC4 (7.1% and 7.0% of the variance, respectively) were influenced exclusively by the JERK parameter.

The four PCs combined highlighted 27 variables of interest.

### Discriminant analysis

**Fig. 4:**
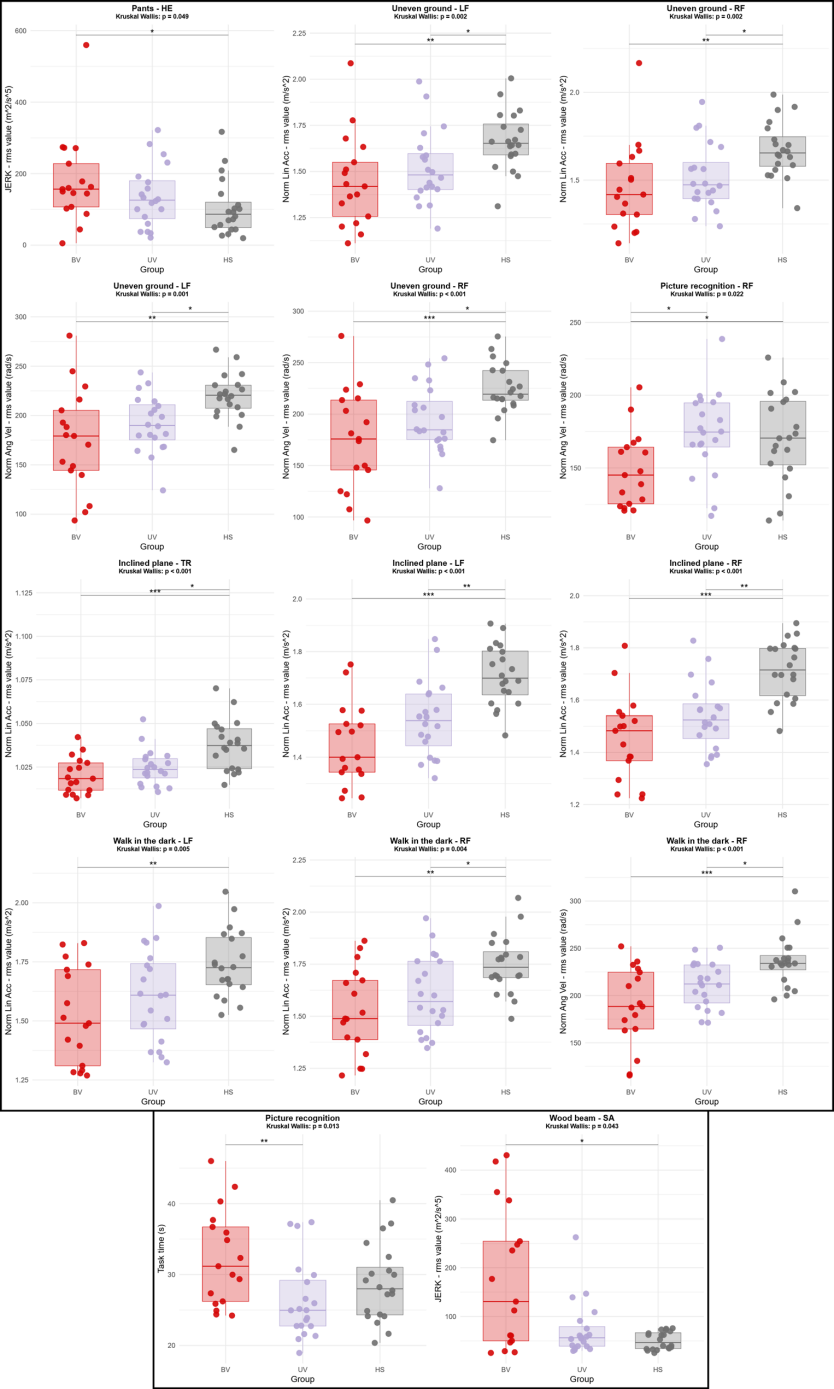
Kruskal-Wallis and Dunn’s post hoc with Holm correction tests for variables extracted from the principal component analysis (PCA). Only parameters showing significant differences are presented. The results of the post hoc test are presented with asterisks: * *p* < 0.05, ** *p* < 0.01, *** *p* < 0.001. Red: Bilateral vestibulopathy patients, Purple: Unilateral vestibulopathy patients, Grey: Healthy subjects. *RF* Right foot, *LF* Left foot, *SA* Sacrum, *HE* Head, *Norm Lin Acc* Linear acceleration norm, *Norm Ang vel* Angular velocity norm, *RMS* root mean square, *BV* Bilateral vestibulopathy *patients*, *UV* Unilateral vestibulopathy *patients*, *HS* Healthy subjects]

Kruskal-Wallis and Dunn’s post hoc tests revealed significant differences between the three groups for 14 of the 27 variables identified in the PCA (Fig. 4). Ten variables came from PC1, two from PC2, and two from PC3. No significant differences were found for variables from PC4.

Six of the eight selected tasks showed significant differences. The Uneven ground task showed the greatest number of differences for the linear acceleration norm (Kruskal-Wallis p-value (KW_p): KW_p = 0.002 and KW_p = 0.002 for LF and RF IMUs, respectively) and for the angular velocity norm (KW_p = 0.001 and KW_p < 0.001 for LF and RF IMUs, respectively). The two other tasks with the greatest number of significant differences were the Inclined plane task with the linear acceleration norm (TR IMU: KW_p < 0.001, LF IMU: KW_p < 0.001, RF IMU: KW_p < 0.001), and the Walk in the dark task with the linear acceleration norm (LF IMU: KW_p = 0.005, RF IMU: KW_p = 0.004) and with the angular velocity norm (RF IMU: KW_p < 0.001). All these variables came from PC1, and Dunn’s post hoc tests (Fig. 4) showed significant differences between the BV-HS and UV-HS groups in each case, with the exception of the linear acceleration norm (LF IMU) for the Walk in the dark task (significant difference between BV-HS only).

During the Picture recognition task, significant differences (Fig. 4) were observed between the BV and UV groups for the task duration parameter, as well as between the BV-UV and BV-HS groups for the angular velocity norm (measured with the RF IMU), KW_p = 0.013 and KW_p = 0.022, respectively. These variables came from PC2.

Regarding variables extracted from PC3, the JERK of the HE IMU for the Pants task and the JERK of the SA IMU for the Wood beam task presented significant differences between the BV and HS groups (Fig. 4), KW_p = 0.049 and KW_p = 0.043, respectively.

## Discussion

This study showed the feasibility of assessing the movements of patients with chronic BV and UV compared with a group of HS using IMUs in a semi-standardized environment. As hypothesized, the results confirmed that tasks reducing the availability of compensatory sensory inputs, thereby increasing vestibular reliance, were the most discriminative between the groups. The most relevant IMUs for quantifying movement were those placed on the feet. This study could serve as a reference for developing reduced and rapid protocols for monitoring the functional status of patients.

The analysis of the duration of the task and the difficulty perceived by the participants enabled the initial number of 15 tasks to be reduced to 7 tasks for the final analysis. An eighth task was added for its clinical relevance, as it is one of the main complaints of patients. These tasks showed significant differences in both the time taken to complete them and the perception of difficulty, reflecting the specific difficulties encountered by patients in their daily lives.

The significantly longer task duration parameter for BV patients compared with HS, and in some cases compared to UV patients, is in line with previous studies [6, 40], suggesting that BV patients may rely on compensatory sensory cues to perform the task more safely and reduce the risk of falling [9, 13, 41]. These patients also reported greater difficulty in performing the task, which could probably be related to a worsening of their symptoms or to the lack of sensory information. This initial selection of tasks confirmed our hypothesis that patients would experience more difficulty performing tasks where compensatory sensory inputs were limited [42, 43]. It is interesting to note that for the “Walk in the dark” task, no significant difference was found in task duration parameter. This result, which is inconsistent with the symptoms and difficulties reported by patients [1, 2], could be explained by the technical limitations of the device used: the glasses were not completely opaque, allowing light to pass through and therefore not reproducing optimal conditions for simulating walking in the dark. It was therefore decided to include this task in the analysis as well to see if any relevant results came up.

The PCA revealed four PCs explaining more than 50% of the total data variance (56%). Only 56% of the total variance was retained because the remaining 44% was explained by several PCs where the percentage of variance per PC was very low.

PC1, composed of 10 variables, mainly explained the intensity of movement during locomotor tasks, with a major contribution from the IMUs placed on the feet. The associated tasks corresponded to locomotor tasks in which complementary sensory inputs (e.g., vision, proprioception) were limited, such as Walking on unstable ground, Walking on an inclined plane, or Walking in the dark. Discriminant analysis between the groups exhibited significantly reduced parameter values compared with those of the HS group, together with greater variability (i.e., a larger inter-quartile range). This suggested that patients may have adopted stabilization strategies by reducing their lower limb movements while walking, particularly in challenging conditions [44]. These adaptations could be explained by several factors: reduced walking speeds [10, 45] leading to longer task durations, but also by affected spatio-temporal parameters, such as increased step width [8, 10], reduced step length [8, 10], or greater variability in walking patterns [8, 13].

PC2, also composed of 10 variables, was characterized by static postural stability tasks such as putting on and taking off pants, and dual tasks such as walking with a heavy load and walking while recognizing pictures. In this PC, the contribution of the IMUs of the upper body segments, i.e. head, trunk, and sacrum, proved to be greater than that of the IMUs of the feet. The highlighted parameters characterized stabilisation (attenuation coefficient), intensity (angular velocity and linear acceleration norms), and smoothness (JERK), probably indicating different motor strategies between pathological groups and HS. These results were consistent with observations regarding increased upper limb rigidity, particularly in the head and trunk in BV patients [12, 13]. Nevertheless, although these variables explained a large part of the variance in the dataset, the discriminant analysis revealed significant differences only for the “Picture recognition” task for right foot angular velocity norm and task duration parameters, suggesting the low necessity of using upper limb IMUs, contrary to what was proposed by Jabri et al. [14]. This major difference could be explained not only by the analysis method used, but also by the protocol and the tasks that were performed.

PC3 and PC4 were only influenced by the JERK parameter, which measures the fluidity of movement, for the pants, wood beam, uneven ground, and inclined plane tasks. However, discriminant analysis showed that only the Pants task (JERK calculated with the head IMU) and the Wood beam task (JERK calculated with the sacrum IMU) showed significant differences between the groups. The JERK values for the BV group were significantly higher than those for the HS group, which contrasted with previous studies that evaluated JERK in patients with Parkinson’s disease [28, 46] or ataxic patients [46], where the JERK values for patients were lower than those for control subjects. In these studies, the values of the control subjects were higher than those of the patients, probably reflecting greater rigidity for patients. This difference between studies could be explained not only by different IMU positions (placed on the upper trunk or upper back), but also by the fact that BV patients, who have more rigid stabilization strategies between the head and trunk [12, 13], may have had larger amplitude movements to compensate for imbalances. In contrast, HS tended to move their trunk less because they had fewer imbalances to compensate for.

The results confirmed the hypotheses that tasks that minimize or deprive compensatory sensory inputs (e.g., proprioceptive or visual cues), thereby increasing reliance on vestibular information, tended to cause the greatest difficulties for patients [42, 43]. Each task presented specific challenges: the Pants task required standing on one leg and coordination to put on and take off the pants, which could cause imbalance due to a shift in the centre of gravity that patients found difficult to adapt to; the “Uneven ground” with stones of different sizes and shapes could cause proprioceptive disturbances and erroneous feedback for maintaining a stable gait without imbalance; the Wood beam task required great balance to put one foot in front of the other without having to catch oneself [11]; the Inclined plane, particularly the descent with eyes closed, disrupted spatial orientation because patients lost their bearings and the compensatory strategies they had put in place; as did the Walk in the dark task, where patients had less visual information to guide them; the Picture recognition task forced patients to rotate their heads while walking without stopping, defeating the head-trunk stiffening strategies they had put in place to compensate for the absent vestibular input that normally informs about head rotations. For this task, it is also possible that patients with oscillopsia were at a disadvantage, although this was not evaluated. As for the hypothesis regarding the best IMUs for assessing movement, this has been partially validated, as it would appear that the use of IMUs on the feet would allow for better differentiation between groups during locomotor tasks. Nevertheless, the IMUs placed on the head, trunk, and sacrum still seemed relevant for tasks requiring static balance (Pants, Wood beam). A multi-sensor approach would allow for a more comprehensive functional assessment, capturing both dynamic locomotor patterns and postural control strategies, but the use of a single sensor on one foot could already provide relevant information.

The suggestion by Jabri et al. [14] to use one single IMU on the left arm was not addressed by our study, as we excluded arm IMUs from the analysis. Future investigations could explore this potential asymmetry in IMUs use.

Finally, the differences between BV and UV are limited, probably due to the heterogeneity of the group of patients with UV, a well-documented but still poorly understood phenomenon [47].

This study had several limitations. First, the sample size was relatively small, with 20 participants per group, due to the availability of patients meeting the selection criteria of the Bárány Society and the geographical recruitment in the French-speaking part of Switzerland. Although a sample size calculation was performed beforehand on a different dataset, including data acquired in a specialized gait laboratory and on the margin of stability parameter [11], and a minimum sample size of 16 participants per group was suggested for sufficient statistical power, further studies should assess the validity, reproducibility, sensitivity, and responsiveness to changes in the tasks, IMUs, and parameters proposed. Secondly, although the semi-standardized environment was more ecological than traditional gait laboratories, it remained supervised and may not have fully reflected the patients’ daily conditions. The patients also really wanted to perform the task well, even though we asked them to perform the task naturally, because they were observed. This behaviour could be attributed to the “Hawthorne effect”, a phenomenon describing the tendency of patients to change the way their symptoms are expressed when they know they are being observed by a healthcare professional [48]. The interactions between patients and operators during task performance, as well as the occurrence of extraneous movements, necessitated the exclusion of certain IMUs. This reduction from 9 to 5 IMUs was motivated by the fact that unwanted movements could introduce noise that could distort the evaluation of task-specific movement patterns. In addition, for practical reasons, this reduction simplified data processing, reduced the parameter computational time, and aligned with similar configurations used in previous studies. The protocol was also conducted over several months of the year, with varying weather conditions that could introduce measurement bias. The development of home monitoring tools thus represents a promising prospect for longitudinal patient assessment [41]. Another limitation lies in the choice of parameters considered (acceleration, jerk), which, although easy to calculate, may not optimally reflect the complexity of postural control. Finally, patient performance levels were quite heterogeneous, due to varying degrees of regular physical activity and variable vestibular physiotherapy follow-up. These parameters were not analysed in the study and could constitute a bias in the results.

The development of a rapid test to monitor patients’ functional status could therefore focus on these six key tasks, with the implementation of a reduced device setup with two IMUs on the feet (or five if the protocol allows). Regarding the calculated parameters, which are easier to implement during the data processing stages, the results suggested focusing on task duration, intensity parameters (linear acceleration norm, angular velocity norm) or fluidity parameters (JERK). This could also help in evaluating the effectiveness of rehabilitation therapies such as vestibular physiotherapy or vestibular implants [16], outside of conventional laboratories [41]. However, this test should be supplemented by conventional clinical assessments and questionnaires.

## Conclusion

This study demonstrated the feasibility of functional and objective assessment of bilateral and unilateral vestibulopathy patients using inertial measurement units in a semi-standardized environment. The identification of 14 discriminating variables (including 6 tasks, 5 IMUs, 4 parameters), which are related mainly to the intensity of movements during locomotor tasks, has opened up promising prospects for the development of rapid clinical assessment protocols. These results also provide preliminary information for functional assessment correlated with patients’ symptoms and limitations in their daily lives. The results also provide perspectives for application in the machine learning field to enable automatic classification, longitudinal patient monitoring, and evaluations of rehabilitation therapies such as vestibular implants.

## Supplementary Information

Supplementary Material 1.

Supplementary Material 2.

Supplementary Material 3.

## Data Availability

The data presented in this study can be found in the following data descriptor article: Grouvel et al., (Grouvel, G., Armand, S., Ghavami, S. et al. Inertial data of daily living tasks in bilateral and unilateral vestibulopathy patients and controls. Sci Data 12, 1649 (2025). https://doi.org/10.1038/s41597-025-05943-4)

## Abbreviations

BV: Bilateral Vestibulopathy
UV: Unilateral Vestibulopathy
HS: Healthy Subject
vHIT: video Head Impulse Test
VOR: Vestibulo- Ocular Reflex
VCR: Vestibulo-Cervical Reflex
IMU: Inertial Measurement Unit
BPPV: Benign Paroxysmal Positional Vertigo
PCA: Principal Component Analysis
PC: Principal Component
sd: standard deviation
HE: Head *IMU*
TR: Trunk *IMU*
SA: Sacrum *IMU*
RF: Right Foot *IMU*
LF: Left Foot *IMU*
RMS: Root Mean Square
AC: Attenuation Coefficient
VAF: Variance Accounted For
KW_p: Kruskal-Wallis p-*value*
