# Supplementary material for: Assessment of dynamic stability and identification of key tasks, inertial sensors, and parameters in patients with bilateral and unilateral vestibulopathy: investigation in a semi-standardized environment
Source: J Neuroeng Rehabil. 2026 Mar 12;23:133. doi: 10.1186/s12984-026-01933-8 (PMC13097859; doi:10.1186/s12984-026-01933-8)
Supplement: Supplementary file 1 — Supplementary Material 1. [file 12984_2026_1933_MOESM1_ESM.docx]

Supplementary Material S1: Raw data of the clinical vestibular tests. vHIT gain and caloric response (if needed) for each patient. BV: Bilateral vestibulopathy; UV: Unilateral vestibulopathy; NR: Not relevant; vHIT: video Head Impulse Test.

| **Patient** | **Group** | **Affected side** | **vHIT gain** | | | | | | **Caloric response °/s** (*if needed*) | | | | | |
| --- | --- | --- | --- | --- | --- | --- | --- | --- | --- | --- | --- | --- | --- | --- |
|  |  |  | **Left side** | | | **Right side** | | | **Left** | | | **Right** | | |
|  |  |  | **Lateral**  **canal** | **Anterior canal** | **Posterior canal** | **Lateral**  **canal** | **Anterior canal** | **Posterior canal** | **Warm water** | **Cold water** | **Warm water** | | **Cold water** | |
| Participant_01 | BV | NR | 0.33 | 0.58 | 0.30 | 0.15 | 0.58 | 0.62 |  |  |  | |  | |
| Participant_02 | BV | NR | 0.77 | 0.58 | 0.65 | 0.15 | 0.50 | 0.48 | 2.3 | 3.4 | 0.5 | | 0.2 | |
| Participant_03 | BV | NR | 0 | 0 | 0 | 0.11 | 0 | 0 |  |  |  | |  | |
| Participant_04 | BV | NR | 0 | 0 | 0 | 0 | 0 | 0 |  |  |  | |  | |
| Participant_05 | BV | NR | -0.05 | - | -0.01 | -0.03 | - | -0.05 |  |  |  | |  | |
| Participant_06 | BV | NR | 0 | 0 | 0 | 0 | 0 | 0 |  |  |  | |  | |
| Participant_07 | BV | NR | 0.67 | 0.72 | 0.37 | -0.01 | 0.75 | 0.37 | -4.5 | 5.0 | -2.9 | | 4.0 | |
| Participant_08 | BV | NR | 0.62 | 0.68 | 0.13 | 0.63 | 0.35 | 0.40 | 0.0 | 0.0 | 0.0 | | 4.8 | |
| Participant_09 | BV | NR | 0.22 | 0.50 | -0.04 | 0.04 | 0.74 | 0.01 |  |  |  | |  | |
| Participant_10 | BV | NR | 0 | 0.35 | 0.18 | 0.03 | 0.30 | 0.40 |  |  |  | |  | |
| Participant_11 | BV | NR | 0.04 | 0.26 | 0 | 0.28 | 0.27 | 0.02 |  |  |  | |  | |
| Participant_12 | BV | NR | 0 | 0.16 | 0 | 0 | 0.18 | 0 |  |  |  | |  | |
| Participant_13 | BV | NR | 0.04 | 0 | 0 | 0 | 0.01 | 0 |  |  |  | |  | |
| Participant_14 | BV | NR | 0.28 | 0.71 | 0.59 | 0.07 | 0.55 | 0.67 |  |  |  | |  | |
| Participant_15 | BV | NR | 0 | 0.36 | -0.05 | 0.74 | 0.29 | -0.03 | 0.0 | 0.0 | 0.0 | | 0.0 | |
| Participant_16 | BV | NR | -0.05 | 0.54 | -0.05 | 0.47 | 0.53 | 0 |  |  |  | |  | |
| Participant_17 | BV | NR | 0 | 0 | 0 | 0 | 0.06 | 0 |  |  |  | |  | |
| Participant_18 | BV | NR | 0.33 | 0.29 | -0.03 | 0.33 | 0.34 | 0.11 |  |  |  | |  | |
| Participant_19 | BV | NR | 0.14 | 0.10 | -0.05 | -0.03 | 0.12 | -0.05 |  |  |  | |  | |
| Participant_20 | UV | L | 0.26 | 0.87 | 0.52 | 1.01 | 0.90 | 0.70 |  |  |  | |  | |
| Participant_21 | UV | L | 0.16 | 0.79 | 0.93 | 1.11 | 1.09 | 0.88 |  |  |  | |  | |
| Participant_22 | UV | R | 0.93 | 0.95 | 0.81 | 0.38 | 0.66 | 0.80 |  |  |  | |  | |
| Participant_23 | UV | L | 0.09 | 0.50 | 0.92 | 0.91 | 0.76 | 0.83 |  |  |  | |  | |
| Participant_24 | UV | R | 0.86 | 0.54 | 0.76 | 0.57 | 0.34 | 0.63 |  |  |  | |  | |
| Participant_25 | UV | L | 0.06 | 0.83 | 0.60 | 0.98 | 0.92 | 0.86 |  |  |  | |  | |
| Participant_26 | UV | L | 0.06 | -0.05 | 0.00 | 0.63 | 1.01 | 0.20 |  |  |  | |  | |
| Participant_27 | UV | L | 0.37 | 0.77 | 0.87 | 1.05 | 0.91 | 0.89 |  |  |  | |  | |
| Participant_28 | UV | R | 1.02 | 1.02 | 0.80 | 0.57 | 0.86 | 0.85 |  |  |  | |  | |
| Participant_29 | UV | L | Not available | | | | | | | | | | |  |
| Participant_30 | UV | R | 0.93 | 1.02 | 0.89 | 0.33 | 0.94 | 0.86 |  |  |  | |  | |
| Participant_31 | UV | L | 0.07 | 0.96 | 0.43 | 0.96 | 1.04 | 0.61 |  |  |  | |  | |
| Participant_32 | UV | R | 0.87 | 0.87 | 0.66 | 0.39 | 0.72 | 0.53 |  |  |  | |  | |
| Participant_33 | UV | R | 0.91 | 0.33 | 0.71 | 0.33 | 0.51 | 0.71 |  |  |  | |  | |
| Participant_34 | UV | R | 0.85 | 0.72 | 0.70 | 0.24 | 0.62 | 0.39 |  |  |  | |  | |
| Participant_35 | UV | R | 1.00 | 1.17 | 0.88 | 0.49 | 1.09 | 0.82 |  |  |  | |  | |
| Participant_36 | UV | R | 0.85 | 0.66 | 0.57 | -0.03 | 0.11 | 0.56 |  |  |  | |  | |
| Participant_37 | UV | R | 0.95 | 1.00 | 0.91 | 0.14 | 0.85 | 0.97 |  |  |  | |  | |
| Participant_38 | UV | L | 0.13 | 0.35 | 0.56 | 0.74 | 0.53 | 0.50 |  |  |  | |  | |
| Participant_39 | UV | R | 0.94 | 1.01 | 0.85 | 0.22 | 0.82 | 0.96 |  |  |  | |  | |
