# Supplementary material for: Assessment of dynamic stability and identification of key tasks, inertial sensors, and parameters in patients with bilateral and unilateral vestibulopathy: investigation in a semi-standardized environment
Source: J Neuroeng Rehabil. 2026 Mar 12;23:133. doi: 10.1186/s12984-026-01933-8 (PMC13097859; doi:10.1186/s12984-026-01933-8)

Results of the Kruskal-Wallis statistical test for task duration and perceived difficulty parameters, followed by the results of Dunn's post-hoc test with Holm correction. ^├^ tasks included in the PCA, * p < 0.05; ** p < 0.01; *** p < 0.001.

| **Task** | **Parameter** | **Kruskal-Wallis (p-value)** | **Post-Hoc Dunn – Holm correction (p-value)** | | | |
| --- | --- | --- | --- | --- | --- | --- |
|  |  |  | **BV - HS** | **UV - HS** | **BV - UV** |  |
| Bed | *Task duration* | 0.007** | 0.006** | 0.327 | 0.072 |  |
|  | *Perceived difficulty* | 0.576 | - | - | - |  |
| Pants^├^ | *Task duration* | 0.007** | 0.009** | 0.039* | 0.480 |  |
|  | *Perceived difficulty* | < 0.001*** | < 0.001*** | < 0.001*** | 0.179 |  |
| Shoes | *Task duration* | 0.095 | - | - | - |  |
|  | *Perceived difficulty* | < 0.001*** | < 0.001*** | 0.025* | 0.049* |  |
| Sorting | *Task duration* | 0.124 | - | - | - |  |
|  | *Perceived difficulty* | 1.000 | - | - | - |  |
| Heavy load^├^ | *Task duration* | 0.028* | 0.022* | 0.188 | 0.345 |  |
|  | *Perceived difficulty* | 0.011* | 0.010** | 0.358 | 0.089 |  |
| Bus | *Task duration* | 0.057 | - | - | - |  |
|  | *Perceived difficulty* | 0.338 | - | - | - |  |
| Stairs | *Task duration* | 0.057 | - | - | - |  |
|  | *Perceived difficulty* | 0.098 | - | - | - |  |
| Uneven ground^├^ | *Task duration* | 0.001** | < 0.001*** | 0.025* | 0.264 |  |
|  | *Perceived difficulty* | 0.005** | 0.004** | 0.052 | 0.328 |  |
| Tray | *Task duration* | 0.331 | - | - | - |  |
|  | *Perceived difficulty* | 0.046* | 0.050* | 0.565 | 0.142 |  |
| Walk | *Task duration* | 0.631 | - | - | - |  |
|  | *Perceived difficulty* | 0.575 | - | - | - |  |
| Stepladder^├^ | *Task duration* | 0.004** | 0.003** | 0.301 | 0.049* |  |
|  | *Perceived difficulty* | 0.022* | 0.025* | 0.095 | 0.497 |  |
| Wood beam^├^ | *Task duration* | < 0.001*** | < 0.001*** | 0.014* | 0.008** |  |
|  | *Perceived difficulty* | < 0.001*** | < 0.001*** | < 0.001*** | 0.196 |  |
| Inclined plane^├^ | *Task duration* | < 0.001*** | < 0.001*** | 0.001** | 0.081 |  |
|  | *Perceived difficulty* | < 0.001*** | < 0.001*** | 0.006** | 0.036* |  |
| Picture recognition^├^ | *Task duration* | 0.013* | 0.179 | 0.304 | 0.019* |  |
|  | *Perceived difficulty* | 0.044* | 0.041* | 0.232 | 0.367 |  |
| Walk in the dark^├^ | *Task duration* | 0.089 | - | - | - |  |
|  | *Perceived difficulty* | 0.006** | 0.005** | 0.140 | 0.179 |  |


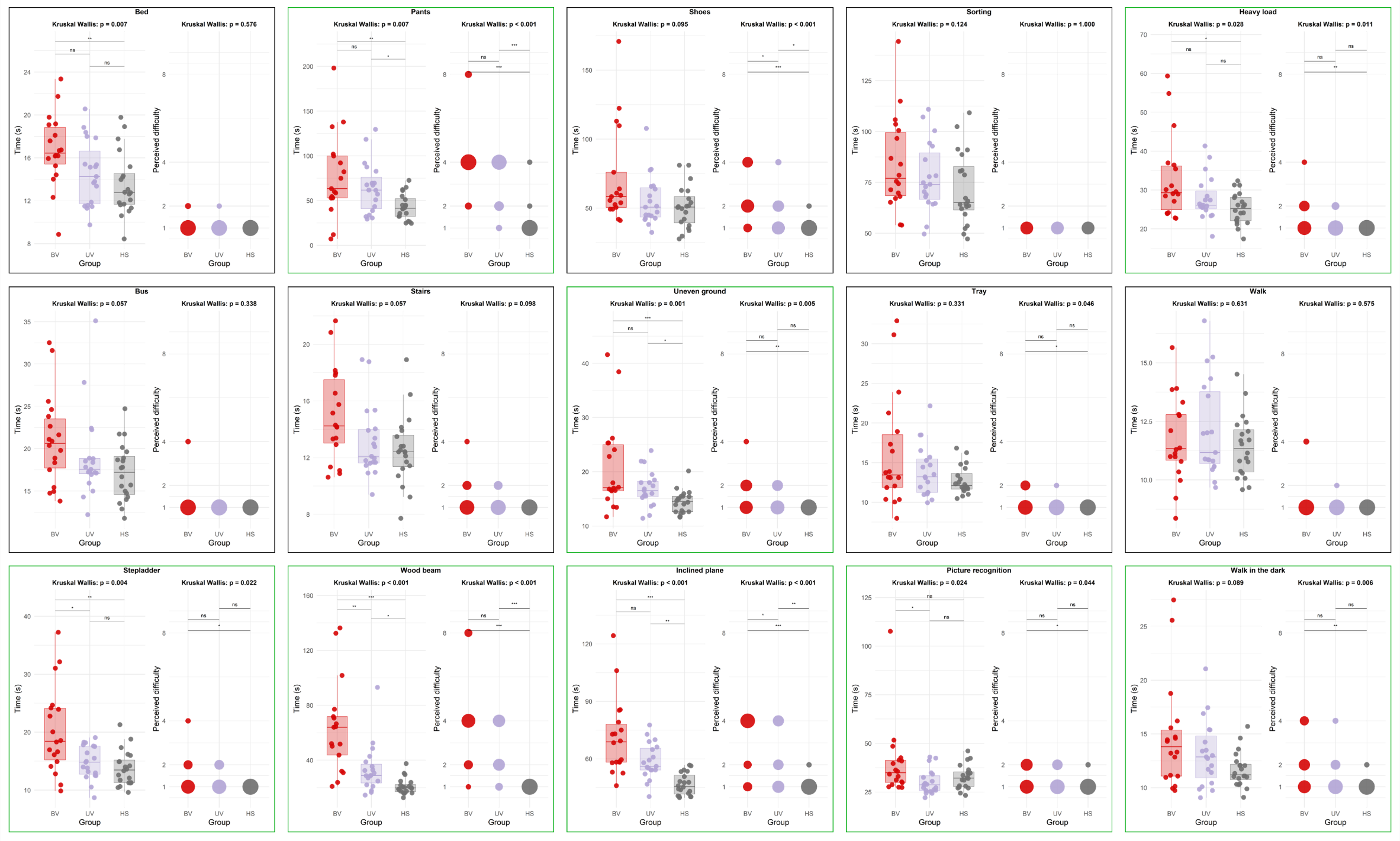

Supplement: Supplementary file 2 — Supplementary Material 2. [file 12984_2026_1933_MOESM2_ESM.docx]
