# Supplementary material for: Assessment of dynamic stability and identification of key tasks, inertial sensors, and parameters in patients with bilateral and unilateral vestibulopathy: investigation in a semi-standardized environment
Source: J Neuroeng Rehabil. 2026 Mar 12;23:133. doi: 10.1186/s12984-026-01933-8 (PMC13097859; doi:10.1186/s12984-026-01933-8)

A. Results of the permutation test to determine how many components to keep. B. Variance explained by principal component.


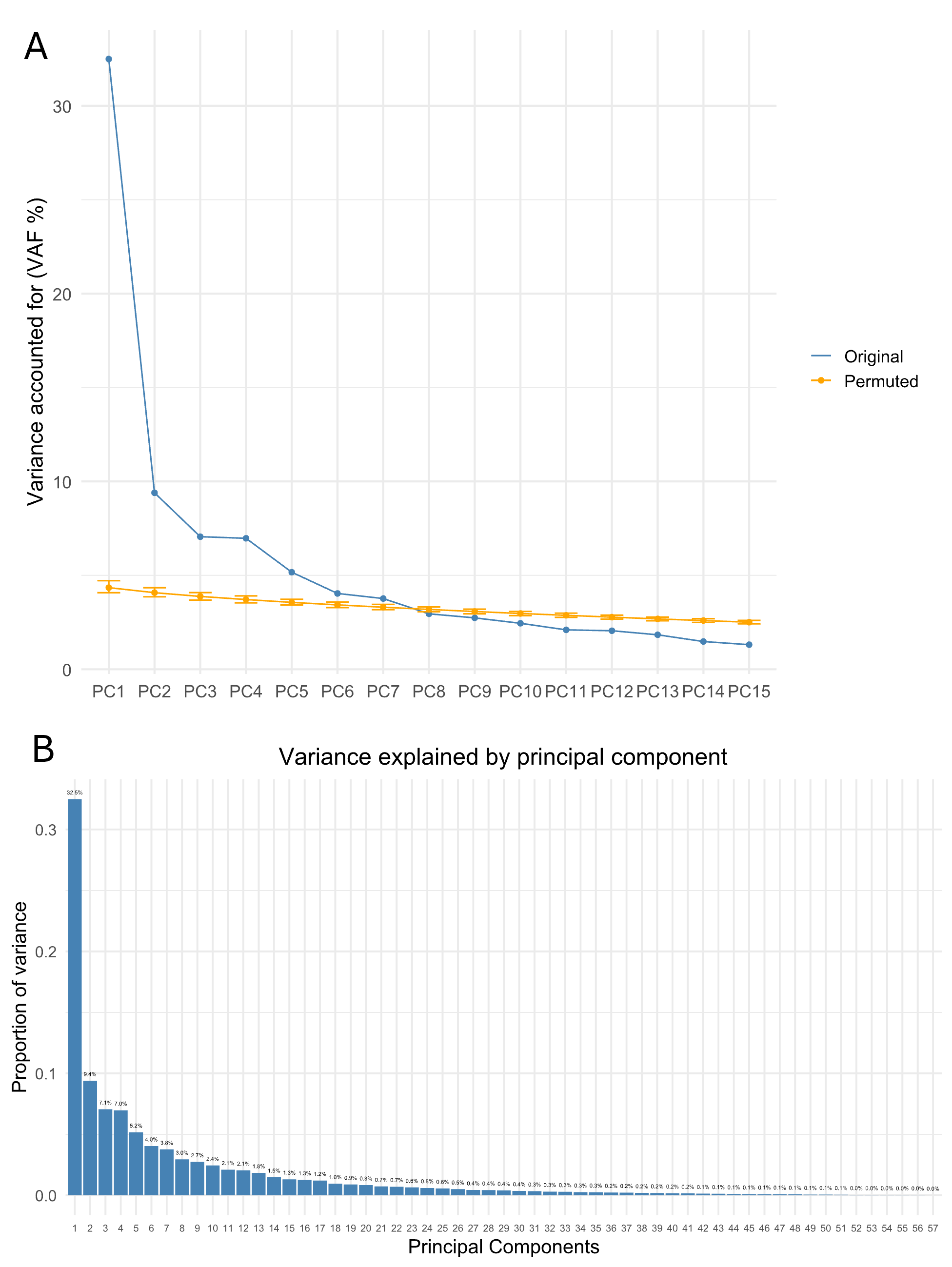

Supplement: Supplementary file 3 — Supplementary Material 3. [file 12984_2026_1933_MOESM3_ESM.docx]
